# Cooperation between BRCA1 and vitamin D is critical for histone acetylation of the p21waf1 promoter and for growth inhibition of breast cancer cells and cancer stem-like cells

**DOI:** 10.18632/oncotarget.2582

**Published:** 2014-11-26

**Authors:** Itay Pickholtz, Shira Saadyan, Gilmor I. Keshet, Victor S. Wang, Rachel Cohen, Peter Bouwman, Jos Jonkers, Stephen W. Byers, Moshe Z. Papa, Ronit I. Yarden

**Affiliations:** ^1^ Laboratory of Genomic Applications, Department of Surgical Oncology, Sheba Medical Center, Ramat-Gan 52621, Israel; ^2^ Sheba Cancer Research Center, Sheba Medical Center, Ramat-Gan 52621, Israel; ^3^ Sackler school of Medicine, Tel Aviv University, Tel Aviv 69978, Israel; ^4^ Department of Human Science, Georgetown University Medical Center, Washington DC 20057, USA; ^5^ Division of Molecular Pathology and Cancer Genomic Center, The Netherland Cancer Institute, Amsterdam 1066, The Netherlands; ^6^ Lombardi Comprehensive Cancer Center, Georgetown University Medical Center, NW Washington DC 20057, USA

**Keywords:** vitamin D, BRCA1, vitamin D receptor, p21waf1, breast cancer, stem cells, histone acetylation

## Abstract

Carriers of germline mutations in the *BRCA1* gene have a significant increased lifetime risk for being diagnosed with breast cancer. The incomplete penetrance of *BRCA1* suggests that environmental and/or genetic factors modify the risk and incidence among mutation carriers. Nutrition and particular micronutrients play a central role in modifying the phenotypic expression of a given genotype by regulating chromatin structure and gene expression. The active form of vitamin D, 1α,25-dihydroxyvitamin D_3_, is a potent inhibitor of breast cancer growth. Here we report that two non-calcemic analogues of 1α,25-dihydroxyvitamin D_3_, seocalcitol (EB1089) and QW-1624F2-2, collaborate with BRCA1 in mediating growth inhibition of breast cancer cells and breast cancer stem-like cells. EB1089 induces a G1/S phase growth arrest that coincides with induction of p21waf1 expression only in BRCA1-expressing cells. A complete knockdown of BRCA1 or p21waf1 renders the cells unresponsive to EB1089. Furthermore, we show that in the presence of ligand, BRCA1 associates with vitamin D receptor (VDR) and the complex co-occupies vitamin D responsive elements (VDRE) at the *CDKN1A* (p21waf1) promoter and enhances acetylation of histone H3 and H4 at these sites. Thus, BRCA1 expression is critical for mediating the biological impact of vitamin D_3_ in breast tumor cells.

## INTRODUCTION

Epidemiological studies carried out over the past several decades suggest that low blood levels of vitamin D are associated with increased risk of several cancer types including breast tumors. Conversely, higher blood levels of vitamin D are associated with reduced risk for developing cancer and with improved prognosis for breast cancer patients [1].

The biologically active form of vitamin D_3_, ((calciferol), 1⍺,25-Dihydroxyvitamin D_3_ (1,25(OH)_2_D_3_)), is a fat soluble hormone and a micronutrient that is obtained either exogenously through diet or by endogenous synthesis following ultraviolet radiation usually in the form of sunlight exposure [2]. In addition to its major role in calcium and phosphate homeostasis, 1,25(OH)_2_D_3_ is a potent inhibitor of cell growth and regulates differentiation, as well as, apoptosis in many types of normal and cancer cells [1].

Vitamin D_3_ exerts most of its cellular effects via its nuclear receptor, the vitamin D_3_ receptor (VDR), that heterodimerizes with the retinoid X receptor (RXR). The VDR-RXR complex binds vitamin D responsive elements (VDRE) in gene promoters and enhancers throughout the genome and regulates transcription of target genes [3]. Upon ligand binding, the VDR-RXR receptor complex undergoes conformational changes leading to the displacement of transcriptional co-repressors with co-activators that enable activation of various biological responses including chromatin accessibility and transcription of specific genes.

The ability of vitamin D_3_ to modulate the genomic landscape and inhibit proliferation of numerous cancer types including prostate, colon and breast cancer has created interest in its potential use as a preventive and/or therapeutic agent. While the lack of a prospective randomized clinical trial is prohibiting a definitive conclusion regarding vitamin D interventions, whether individuals with certain genetic profiles will benefit more or less from vitamin D-based therapies has been overlooked.

*BRCA1* is the most frequently mutated tumor suppressor gene in breast cancer [4]. Loss of BRCA1 expression is also associated with an increased risk of several types if cancer [5–7]. BRCA1 is a multifunctional protein involved in many fundamental cellular processes including cell cycle regulation, DNA repair, transcription, chromatin modifications and ubiquitylation, all contributing to its role in maintenance of genomic stability and tumor suppression [8]. The BRCT domain at the C-terminal of BRCA1 was the first functional element identified in the BRCA1 protein important for BRCA1-mediated transactivation [9]. The domain is also known to bind phospho-proteins [10–12] and it is the site for association with the RNA polymerase II holoenzyme [13], transcription factors including p53 [14], DNA helicases such as FANCJ [15], and chromatin modifying enzymes such as HDAC1/HDAC2 [16]. Cancer-associated mutations in the BRCT domain abrogate BRCA1 interaction with these various proteins and impair its transactivation activity [8, 17].

Here we show that BRCA1 expression is also critical for vitamin D_3_-mediated inhibition of ER positive and ER negative breast cancer cell proliferation, as well as that of mammosphere cultures enriched with stem-like cancer cells. We show that the non-calcemic 1,25(OH)_2_D_3_ analogue (EB1089), induces BRCA1 association with VDR and its recruitment to three VDRE sites located in the promoter region of another tumor suppressor gene, *CDKN1A,* to enhance *CDKN1A* expression. *CDKN1A* encodes for the p21waf1 protein, a cell cycle regulator, critical for activation of the G1/S checkpoint under various conditions including exposure to vitamin D_3_. In addition, we show that MCF7 cells depleted for p21waf1 failed to arrest and continued to proliferate in response to EB1089.

Our results reveal a novel aspect of BRCA1 function unrelated to DNA repair. Our data suggest that vitamin D-based therapies or prevention should take into account patient-specific genetic background.

## RESULTS

### Effect of 1,25(OH)_2_D_3_ analogues on growth of BRCA1-deficient and proficient breast cancer cells

**Figure 1:**
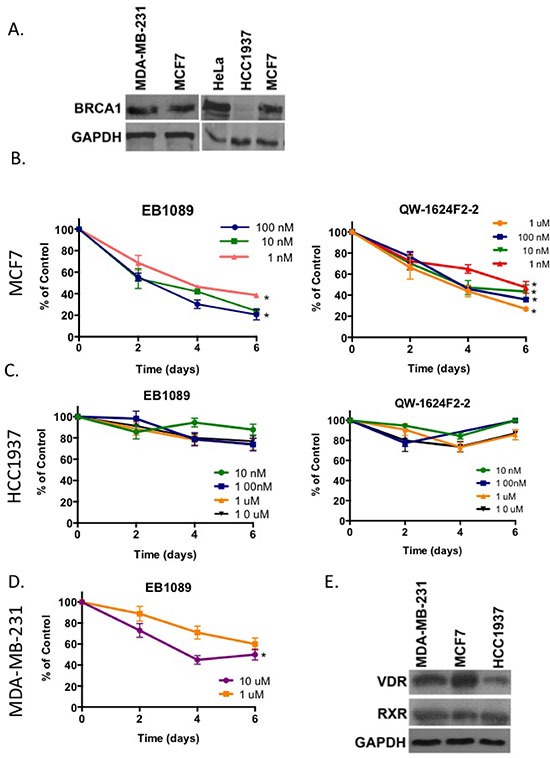
Vitamin D_3_ analogues inhibit growth of BRCA1 expressing breast cancer cells **(A)** 100 μg of the total cell lysates isolated from different cell lines were analyzed for BRCA1 expression by immunoblot analysis. GAPDH was analyzed as a control for equal loading. **(B-D)** MCF7 cells (4000 cells/well) (B), HCC1937 cells (4000 cells/well) (C) and MDA-MB-231 cells (1500 cells/well) (D) were plated in 6 replicates in 96 well plates. On the following day cells were washed twice with 1x PBS and the growth media was replaced with phenol-red free DMEM supplemented with 10% charcoal-stripped FBS. One day later (*t* = 0), cells were treated with either EB1089 (left panel) or QW-1624F2-2 (right panel) at the indicated concentrations. Cell proliferation was analyzed every 48 hr following crystal violet staining and absorbance measurements at 540nm. Values represent the percentage of growth relative to vehicle (EtOH)-treated cells. Graphs are representative of the mean ± standard deviations (SD) of 6 replicates in three independent experiments. Statistical analysis was performed using two tails Student *t*-test. Significant results indicated *p < 0.04 for EB1089 or QW-1624F2-2 versus vehicle treated cells. **(E)** 50 μg of total cell lysates isolated from MCF7, MDA-MB-231 and HCC1937 were analyzed for VDR and RXR expression by immunoblot analysis.

To examine whether BRCA1 expression correlates with vitamin D_3_ anti-proliferative effects, three breast epithelial cell lines were used as models (Figure 1A). MCF7 is an estrogen responsive, ER positive, adenocarcinoma cell line that expresses wild type BRCA1. MDA-MB-231 is a triple receptor negative metastatic carcinoma cell line that expresses wild type BRCA1. HCC1937 is a BRCA1-null, adenocarcinoma cell line that harbors the 5382insC mutation in the *BRCA1* gene and is ER negative. As the naturally occurring, biologically active form of vitamin D_3_, 1,25(OH)_2_D_3_, causes hypercalcemia at pharmacologically relevant doses and it cannot be clinically used, we tested two different non-toxic analogues of vitamin D_3_, EB1089 and QW-1624F2-2 [18, 19] for their growth inhibitory effects on the three cell lines. Cells were depleted of estrogen by replacement of the culture media with phenol-red free DMEM supplemented with 10% charcoal-treated serum. A time course and a dose-response ranging from 0.1 nM–10 μM demonstrated that proliferation of MCF7 cells was inhibited by EB1089 and QW-1624F2-2 up to 80% relative to vehicle (EtOH)-treated cells (Figure 1B). HCC1937 cells proliferation was only slightly inhibited (~20% reduction) relative to vehicle-treated cells (Figure 1C). MDA-MB-231 cells showed an intermediate response to EB1089 and their growth was inhibited up to 60% of vehicle-treated cells, albeit a higher concentration was needed (Figure 1D). Overall, EB1089 (IC_50_ of 3 × 10^−9^ M in MCF7) was more potent than QW-1624F2-2 (IC_50_ of 1 × 10^−8^ M) when calculated for cells treated over the course of 6 days, and was chosen for further studies. Immunoblot analysis suggests that VDR protein expression levels are lower in MDA-MB-231 and HCC1937 cells than in MCF7 cells while RXR expression is similar among the different cell lines. Yet, differences in responsiveness to vitamin D could not be solely attributed to differences in VDR expression (Figure 1E).

### Effect of 1,25(OH)_2_D_3_ analogue, EB1089, on growth of BRCA1-silenced breast cancer cells

**Figure 2:**
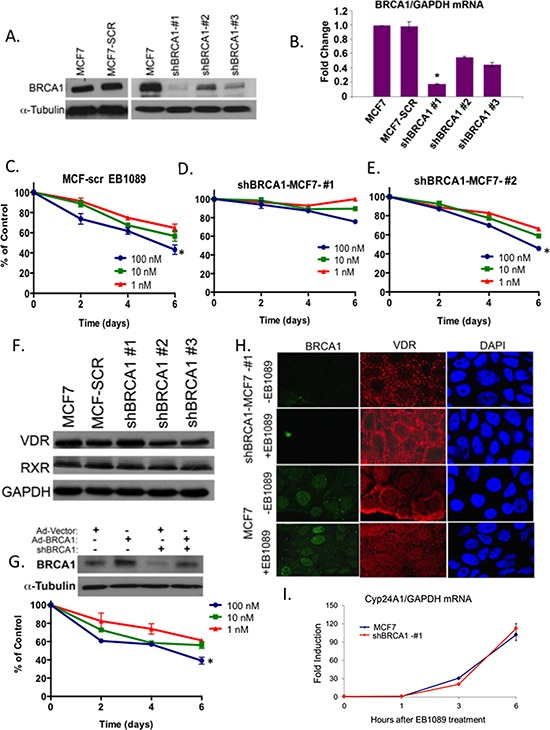
BRCA1 expression levels are critical for EB1089-mediated growth inhibition **(A)** MCF7 cells were stably infected with a cocktail of SMARTpool shBRCA1 or with shScrambled sequence of BRCA1. Puromycin–resistance colonies were expanded and 100 μg of whole cell lysates from the newly formed sublines were analyzed for BRCA1 expression by immunoblot analysis. **(B)** Total RNA was extracted from cells in (A) and BRCA1 mRNA expression was analyzed relative to GAPDH in three replicates by qRT-PCR in three independent experiments. Statistical analysis was performed using a two-tailed Student *t*-test. *p < 0.01 versus MCF7 cells. **(C-E)** Three MCF7 sublines expressing very low (shBRCA1-1), intermediate (shBRCA1-2) and normal levels of BRCA1 (MCF-SCR) were seeded into 96 well plates (4000 cells/well) and their growth in response to the indicated concentrations of EB1089 was analyzed by crystal violet assay. Values represent the percent of control, vehicle treated cells. Graphs are representative of the mean ± SD of 6 replicates in three independent experiments. Statistical analysis was performed using a two-tailed Student *t*-test. *p < 0.04 **(F)** 50 μg of whole cell extracts isolated from cells in (A) were analyzed for VDR and RXR expression by immunoblot. **(G)** MCF7 control and shBRCA1#1-MCF7 cells were infected with either Ad-vector or Ad-BRCA1 expression vectors (MOI = 100) as described [57]. After 24 hr, whole cell lysates were isolated and analyzed for BRCA1 expression. Following re-expression of BRCA1, shBRCA1#1-MCF7 cells were seeded into 96 well plates (4000 cells/well). On the following day, media was replaced by phenol-red free DMEM with 10% charcoal-treated FBS. Cells were treated with the indicated concentrations of EB1089 and changes in growth were measured every 48 hr following staining with Crystal Violet. Values represent percentage of control growth of vehicle-treated cells and were calculated as mean ± SD of 6 replicates from two independent experiments. (*p < 0.04) **(H)** Immunofluorescence to detect VDR and BRCA1 in BRCA1-deficient (shBRCA1-#1) and proficient MCF7 cells 30 min after treatment with vehicle or 1 μM of EB1089. Representative pictures from three different experiments are shown. **(I)** MCF7 and shBRCA1-#1-MCF7 cells were treated with 1 μM EB1089 for the indicated times and Cyp24A1 mRNA expression relative to GAPDH was analyzed by qRT-PCR. Fold induction was calculated compared to the untreated cells from three replicates based on ΔΔCT. Statistical analysis of the correlation between MCF7 and shBRCA1-#1-MCF7 cells was done using the Pearson correlation coefficient test, *r* = 0.97, *p* < 0.0001.

To determine the direct role of BRCA1 in vitamin D_3_ mediated growth inhibition, we stably silenced BRCA1 expression in MCF7 and MDA-MB 231 cells using several shRNA constructs. Three stable shBRCA1-MCF7 sub-lines expressing low (MCF7-#1, #3) and intermediate (MCF7-#2) BRCA1 mRNA and protein levels were isolated and characterized as well as a control line expressing scrambled shRNA (MCF7-SCR) (Figure 2A and 2B). Two sublines of MDA-MB-231 cells were isolated (Supplementary Figure S1). A time course and dose-response analyses of the different MCF7 sublines, showed that only cells that express BRCA1 (normal and intermediate levels) were significantly growth inhibited (~60% reduction) by 0.1–1 μM EB1089 relative to vehicle-treated cells. The proliferation of MCF7-shBRCA1#1 cells that were efficiently silenced for BRCA1 (>90% reduction) was not inhibited (Figure 2C, 2D, 2E). Similarly, BRCA1-silenced MDA-MB-231 cells were not inhibited to the same extent as BRCA1-proficient-MDA-MB-231 cells. (Supplementary Figure S2). Immunoblot analysis did not detect significant loss of VDR or RXR expression in BRCA1-silenced cells (Figure 2F). Reconstitution of BRCA1 expression in BRCA1-silent MCF7 cells via transient infection with Ad-BRCA1 [20] partly restored vitamin D_3_ responsiveness and confirmed the role of BRCA1 in vitamin D_3_ growth inhibition (Figure 2G). VDR constantly shuttles between the cytosplasm and the nucleus [21, 22]. Since VDR expression levels are similar in BRCA1 deficient and proficient cells and nuclear translocation of VDR is critical for vitamin D_3_-induced transcription, we investigated whether VDR is mislocalized in BRCA1-deficient cells. Immunofluorescence staining showed that, VDR is prominently cytosolic and also present in the nucleus of control untreated MCF7 cells (Figure 2H). Following EB1089 treatment for 30 min, most VDR translocated into the nucleus as shown by the significant increase in nuclear staining (Figure 2H). In BRCA-silenced cells, a distinct cytoplasmic staining with no nuclear staining of VDR was detected in untreated cells as previously shown [23]. However, following EB1089 treatment, nuclear localization of VDR was observed in a majority of the cells (*n* = 80) (Figure 2H).

To verify that VDR is transcriptionally active in BRCA1-silenced cells, we analyzed whether vitamin D can induce the mRNA expression of its target gene coding for the mitochondrial enzyme 1,25-dihydroxyvitamin D_3_ 24-hydroxylase, CYP24A, a member of the cytochrome P450 family. The enzyme catabolizes vitamin D_3_ by hydroxylation as a negative feedback mechanism for maintaining calcium and vitamin D_3_ homeostasis [24, 25]. A rapid induction of up to 20 fold by 3 hr and about 100 fold by 6 hr of CYP24 mRNA was noted in both MCF7 and BRCA1-silenced MCF7 cells suggesting that VDR is transcriptionally active in BRCA1-silenced MCF7 cells and supporting that vitamin D_3_-growth inhibition is uncoupled from calcium homeostasis (Figure 2I) [26].

### Mammosphere cultures with mammary cancer stem cells properties are growth inhibited by vitamin D

**Figure 3:**
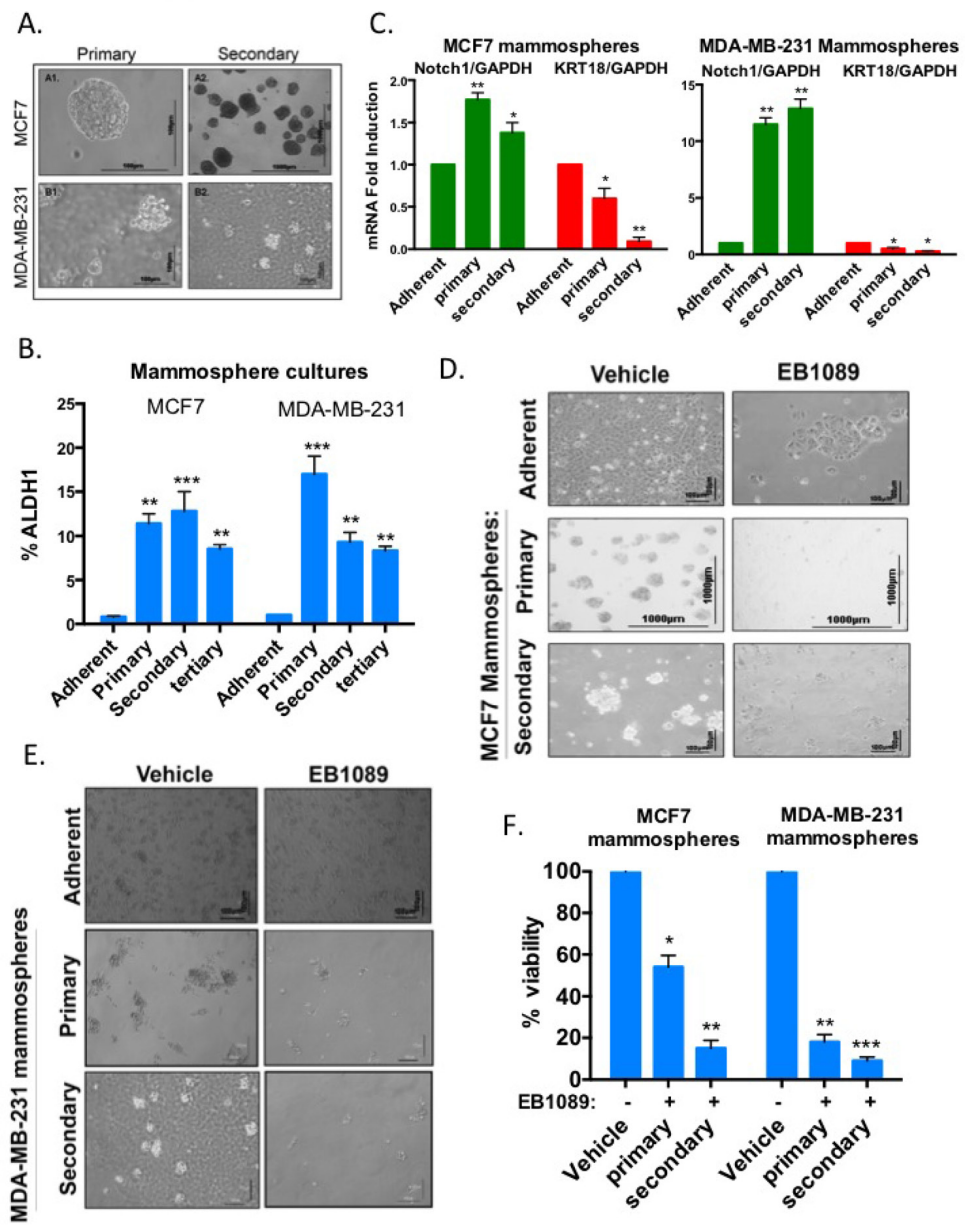
Growth and self-renewal of mammospheres enriched with stem-like breast cancer cells are inhibited by EB1089 **(A)** MCF7 and MDA-MB-231 adherent cells were plated in non-adherent plates in a mammosphere-defined media. After 7 days, primary mammospheres were dissociated and replated in a limiting dilution (1000 cells/6 well plate) into non-adherent plates to form secondary mammospheres. A similar procedure was used to form tertiary mammospheres. Bright field images of MCF7 (upper panel) and MDA-MB-231 (lower panel) of primary (left panel × 20) and secondary mammospheres (right panel × 10) were taken using a NIKON microscope with two different magnifications. Scale bar is 100 μ. **(B)** Analysis of ALDH1 expression in primary, secondary and tertiary MCF7 and MDA-MB-231 mammospheres. Adherent and mammosphere cultures were prepared as single cells suspensions and analyzed according to manufacturer's instructions (Aldefluor kit, Stem Cell Technologies) by flow cytometry. Bar graphs represent the percentage of ALDH positive cells. Student's *t*-test (2-tailed, paired) was used to evaluate mammosphere cultures with adherent cells and regarded as being significant if *p* < 0.05 (*) more significant **p < 0.0007 and with highest significance ***p < 0.0001. **(C)** Total RNA was isolated from adherent, primary and secondary mammosphere cultures derived from MCF7 and MDA-MB-231 cells. mRNA expression of Notch1, a marker for self-renewal, and KRT18, a marker for differentiation, relative to GAPDH was analyzed by qRT-PCR. Bar graphs represent mean ± SD of 3 replicates from 3 experiments. Statistical significance was analyzed by Student's *t*-test (2-tailed, paired) and results regarded significant if *p* < 0.05 (*) or more significant if *p* < 0.03 (**). **(D-E)** Adherent MCF7 (D) or MDA-MB-231 **(E)** cells and primary and secondary mammospheres were cultured in the presence of 0.1 or 1 μM of EB1089. Growth factors and EB1089 were replenished after 3 days. Representative images were taken after 6–7 days when vehicle treated cells reached confluency or maximal growth. **(F)** MCF-7 and MDA-MB-231 primary and secondary mammospheres were treated with vehicle or 1 μM EB1089. 6 days later, cell viability was determined by XTT. Data reported as % of vehicle control. Bars, Mean ± SD of triplicate samples. Student's *t*-test (2-tailed, paired) was used and results regarded significant if *p* < 0.002 (*) or more significant (**p < 0.0002) and highly significant if *p* < 0.0001 (***).

Breast tumors initiate and often recur due to the presence of cancer stem cells within the mammary tissue. These stem cells are refractory/resistant to most conventional chemotherapy and radiation therapy [27, 28]. Thus, successful elimination of tumors will depend on treatments that target mammary cancer stem cells in addition to the bulk of highly proliferative cells. Cancer cell lines can be enriched for cell populations with stem-like properties when grown in suspension as mammospheres in a defined serum-free media supplemented with growth factors [29]. Here we analyzed the effects of vitamin D_3_ analogue (EB1089) on the formation, viability and self-renewal potential of mammosphere cultures derived from BRCA1-expressing MCF7 and MDA-MB-231 cells and BRCA1-silenced MCF and MDA-MB-231 cells. We first enriched for stem-like cells by seeding cells in non-adherent plates in a defined mammosphere media [29, 30]. After 7 days, mammospheres reached approximately size of 100μM (primary mammospheres). The self renewal potential of the mammosphere cultures was evaluated following dissociation of mammospheres into single cell suspensions and re-plating in a limiting dilution into new non-adherent plates with fresh media (Secondary mammospheres). This process was repeated every 7 days. The enzymatic activity of the “stemness” marker, aldehyde dehydrogenase 1, ALDH1 [31], was measured in primary, secondary and tertiary mammosphere cultures by an ALDEFLUOR assay and flow cytometry. The most significant enrichment in ALDH1 activity was noted after the initial formation of mammosphere cultures, at the end of the first week. Activity was mostly sustained in secondary and tertiary mammospheres (Figure 3A and 3B and Figure S3A and S3B). The induction of Notch1, the stemness and self renewal marker, mRNA expression together with the down-regulation of the differentiation marker, keratin 18, were analyzed by qRT-PCR and further confirmed the stemness and self-renewal potential of the primary and secondary mammosphere cultures (Figure 3C).

We then examined whether vitamin D_3_ inhibits mammosphere formation and self-renewal of MCF7 and MDA-MB-231 cells. Cells were seeded in low-attachment plates in the presence of mammosphere-defined culture media with EB1089. Mammospheres were monitored over 7 days and growth factors and EB1089 were replenished every 3 days. Mammospheres were visualized when control spheres reached about 100 μm (about 7 days). A significant reduction in mammosphere formation was noticed in EB1089 treated cells (Figure 3D and 3E). The self-renewal capacity of EB1089-treated mammospheres was significantly reduced as the ability of dissociated primary mammospheres to re-grow and form secondary mammospheres was diminished (Figure 3D and 3E). XTT assays confirmed that EB1089 decreased the viability of MCF7 and MDA-MB-231 mammospheres (Figure 3F).

### BRCA1-silenced mammospheres are no longer growth inhibited by vitamin D

**Figure 4:**
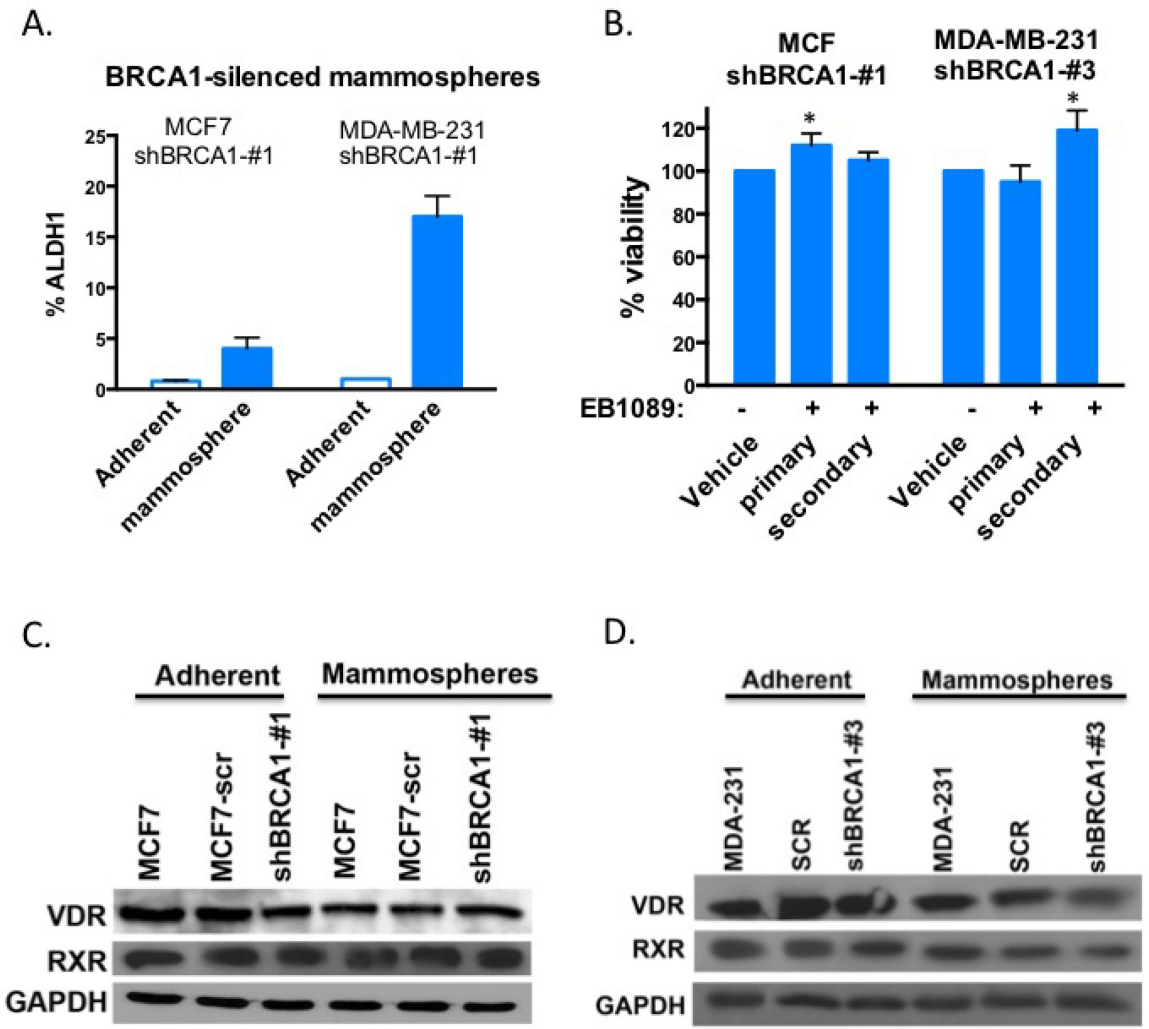
BRCA1-silenced mammospheres are not inhibited by EB1089 **(A)** BRCA1-silenced MCF7 and MDA-MD-231 cells were grown as adherent culture or as mammospheres and ALDH1 activity was determined by the Aldefluor kit (Stem Cell Technologies) and flow cytometry. Bar graphs showing the percentage of ALDH1 positive cells. Student's *t*-test was used to evaluate differences between mammosphere cultures with adherent cells and regarded as being significant if *p* < 0.0075 (*) more significant **p < 0.0002 **(B)** BRCA1-silenced MCF7 and MDA-231-derived mammospheres were grown in non-adherent plates in the presence of a defined media, and treated with either vehicle or 1 μM EB1089. Growth factors and EB1089 were replenished after 3 days. Viability of cells was analyzed by XTT 6 days after the initiation of treatment. Data reported as % of vehicle control. Student's *t*-test was used to evaluate differences between mammosphere cultures with adherent cells and regarded as being significant if *p* < 0.05 **(C-D)** Whole cell lysates were prepared from BRCA1-silenced or proficient adherent MCF7 (C) or MDA-MB-231 (D) and their derived mammospheres cultures and VDR and RXR expression was analyzed by immunoblot analysis.

Mammosphere cultures derived from BRCA1-silenced MCF7 and BRCA1-silenced MDA-MB-231 cells were enriched with ALDH1 activity similar to BRCA1-expressing mammospheres (Figure 4A). While, EB1089 treatment inhibited the formation and viability of BRCA1-proficient mammospheres (Figure 3D-F), BRCA1-deficient MCF7 and MDA-MB-231 formed viable mammospheres in the presence of EB1089 in the culture media, as determined by an XTT assays (Figure 4B). Furthermore, the self-renewal potential of mammospheres derived from BRCA1-silenced cells was not affected significantly by vitamin D_3_ (Figure 4B). We examined the expression of VDR and RXR in MCF7 and MDA-MB-231 cells grown either as adherent cultures or as mammospheres, and in mammosphere cultures that were derived from BRCA1-silenced MCF7 and MDA-MB-231 cells by immunoblot analysis. No significant changes were detected in RXR expression between adherent and mammospheres in either MCF7 or MDA-MB-231 cells that express or do not express BRCA1 (Figure 4C and 4D). VDR expression in MCF7 derived mammospheres was reduced relative to adherent cells, but no difference in expression was noted between those that express BRCA1 or not, suggesting that small changes in VDR expression do not account for differences in response to vitamin D_3_ (Figure 4C).

### BRCA1 modulates the vitamin D - VDR – signaling axis and mediated growth inhibition in Murine cells

**Figure 5:**
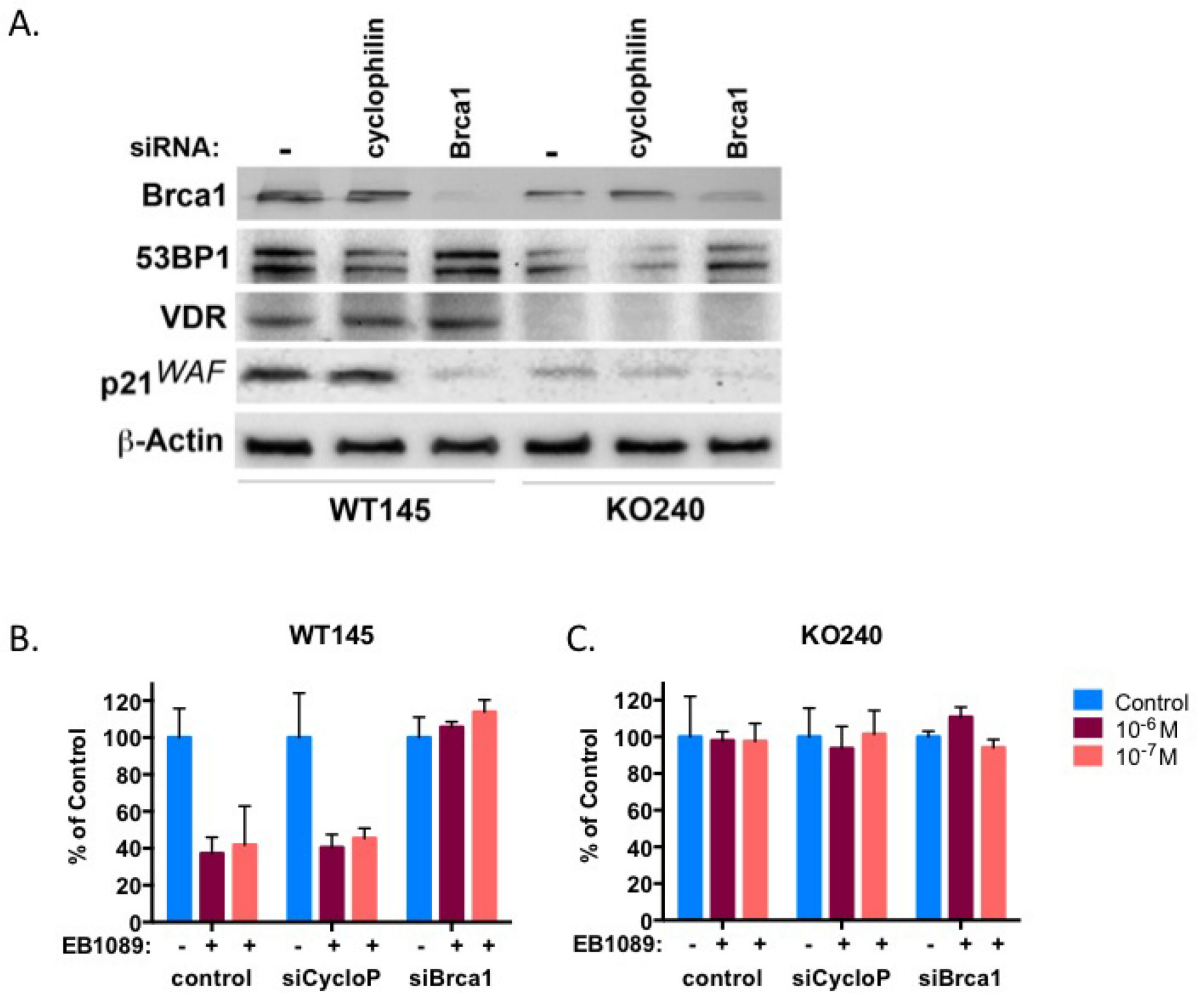
BRCA1 and VDR work in concert in mammary murine cells Normal mammary murine cells, WT145, and isogenic mammary cancer cells, KO240, were transiently transfected with the indicated siRNA and after 72 hr whole cell lysates were prepared and subjected to immunoblot analysis with the indicated antibodies. **(B-C)** WT145 and KO240 cells were seeded in triplicates into 96 well plates and were transfected with the indicated siRNA by reverse transfection. On the following day the culture media was replaced with phenol-red free DMEM/F12 supplemented with 10% charcoal-stripped FBS to which vehicle or different concentrations of EB1089 were added. Viability of cells was determined after 3 days by XTT. Bars, Mean ±SD of 3 replicate samples from three independent experiments.

To further verify that BRCA1 is required for vitamin D_3_-mediated growth inhibition, we used SiGenome siRNA pool to transiently silence Brca1 in two murine isogenic mammary tumor cells, WT145 and KO240, that were previously described [32]. KO240 cells originated from a mammary tumor in VDR-knockout mice, they do not express VDR and are resistant to vitamin D_3_ [32]. *Brca1* knockdown in WT145 and KO240 and lack of VDR expression in KO240 were confirmed 72 hr after transfection by an immunoblot analysis (Figure 5A). Silencing of cyclophillin B was carried out as a non-specific targeting control. Growth inhibition of Brca1-silecnced WT145 and KO240 cells in response to EB1089 or vehicle alone was analyzed by XTT assays and the results corroborate our initial findings that absence of BRCA1 impairs the growth inhibitory response to vitamin D_3_ (Figure 5B and 5C). Vehicle-treated cells and control-silenced cells were growth inhibited by EB1089 similar to parental WT145 cells (Figure 5B). As expected, parental KO240 control did not respond to EB1089 and no response was noted in the Brca1-silenced cells (Figure 5C).

### EB1089 induction of G1 phase cell cycle arrest and the expression of p21/waf is dependent on BRCA1 expression

**Figure 6:**
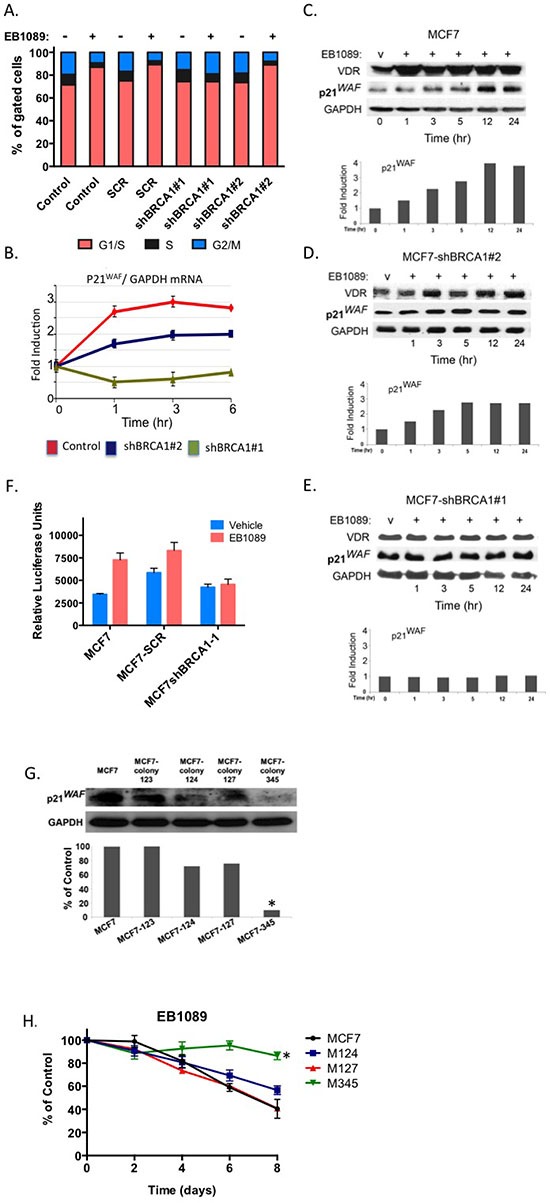
p21waf1 mediates the EB1089-induced growth inhibition in BRCA1-proficient cells **(A)** MCF7 control and stably expressing shRNAs that target BRCA1 were treated with 1 μM of EB1089 or vehicle for 24 hr and analyzed for cell cycle distribution by flow cytometry following staining with propidium iodide. A non-targeting shRNA served as a negative control. Data represent three independent experiments. **(B)** Total RNA was isolated from BRCA1-deficient and proficient MCF7 cells that were treated with vehicle or 1 μM for the indicated times. Expression of p21waf1 mRNA was analyzed relative to GAPDH by qRT-PCR. Results represent mean ± SD of triplicate samples from more than three independent experiments. **(C-E)** MCF7 control (C) and MCF7 stably expressing BRCA1-targeting shRNAs: (D) shBRCA1-#2-MCF7 and (E) shBRCA1-#1-MCF7 were treated with vehicle or 1 μM EB1089 for the indicated times. Whole cell lysates were prepared and subjected to immunoblot analysis for the indicated proteins. Quantification of band intensity corresponding to p21waf1 protein is presented in bar graphs. **(F)** BRCA1-deficient and proficient MCF7 cells were transiently co-transfected with pWW-Luc and a pRL-Renila reporter plasmids and 24 hr later cells were treated with either vehicle or 1 μM of EB1089 for 24 hr. Luciferase expression levels were normalized to Renilla levels. Bar graphs represent mean ±SD of triplicate samples from three independent experiments. **(G)** MCF7 cells were stably transfected with shRNAs targeting p21waf1 and puromycin resistant clones were selected. Whole cell lysates were prepared from the different clones and the expression level of p21waf1 was determined by immunoblot analysis. ImageJ was used for qunatification of band intensity. **(H)** Cell lines from (G) were seeded in 4 replicates into 96 well plates in phenol-red free DMEM with 10% charcoal-treated FBS. On the following day, cells were vehicle or 1 μM of EB1089 and growth was measured every 48 hr by Crystal violet assay. The values represent the percent of growth relative to vehicle treated cells. Statistical analysis was performed using a one-way analysis of variance.*p < 0.008 for M345 and *p* = 0.566 for M124 versus MCF7 cells.

Vitamin D_3_ is known to induce G1/G0 cell cycle arrest [33, 34]. To investigate whether BRCA1 is required for induction of the G1 checkpoint, cells expressing BRCA1-targeting and non-targeting shRNAs (shBRCA1-#1, shBRCA1-#2, MCF7-SCR) and parental MCF7 were treated with EB1089 for 24 hr and then analyzed by propidium iodide staining and flow cytometry. As expected, BRCA1-expressing MCF7 cells accumulated at the G1/G0 phase of the cell cycle in response to EB1089. However, BRCA1-silenced cells failed to accumulate at any phase of the cell cycle. No apoptotic cells at the subG1 phase were detected (Figure 6A).

The G1 phase cyclin-dependent kinase inhibitors, p21waf1 and p27kip, are known targets of vitamin D_3_ signaling [34]. qRT-PCR analysis of p21waf1 mRNA in parental, partially-silenced BRCA1- and BRCA1-silenced MCF7 cells following treatment with EB1089 demonstrated that p21waf1 mRNA was induced (up to 3 fold) in BRCA1-expressing cells (Figure 6B). p21waf1 mRNA expression was modestly induced in partially-silenced MCF7 cells that express about half of the amount of BRCA1 protein. No change in p21waf1 was detected in BRCA1-silenced MCF7 cells (shBRCA1-#1-MCF7 cells). p21waf1 protein expression correlated tightly with mRNA expression levels. (Figure 6C, 6D and 6E). Furthermore, silencing of Brca1 expression in wild type, WT145+/+ murine cells, down regulated the expression levels of p21waf1 protein (Figure 5A). The basal expression of p21waf1 in VDR knockout cells (KO240 cells) is lower than the level detected in WT145 cells. Silencing of Brca1 in VDR knockout cells slightly reduced p21waf1 expression (Figure 5A). To further confirm the direct transcriptional activation of p21waf1 expression by vitamin D_3_ and BRCA1, we transfected the pWWP-Luc reporter that contains the proximal promoter of *CDKN1A* gene [kind gift of K. Kinzler, JHU] to BRCA1-depleted and control MCF7 cells. Only a modest response of less than 2.0 fold induction was seen in parental MCF7 cells, which might be a result of the presence of only the proximal promoter sequence. No induction of luciferase was seen in the BRCA1-silenced cells (Figure 6F). Taken together, these data show that BRCA1 expression is important for p21waf1 induction by EB1089, although additional sequences in the *CDKN1A* gene may contribute to vitamin D_3_ induction.

Conversely, p27 protein expression was not altered significantly in the cell lines examined following 24 hr treatment with EB1089 (Supplementary Figure S4A-C). However, we noted a significant increase in p27 expression 48 and 96 hr following EB1089 treatment (Supplementary Figure S4D). Taken together, this data suggest that vitamin D_3_ and BRCA1 work in concert to transcriptionally up regulate their mutual target gene, *CDKN1A,* while up regulation of p27 (*CDKN1B*) expression is probably co-regulated indirectly by vitamin D_3_ and BRCA1.

To determine whether p21waf1 plays a role in vitamin D_3_-mediated growth inhibition, we stably silenced p21waf1 expression in MCF7 cells by different cocktails of p21waf1-specific shRNAs (Supplementary Table 2). Several resultant cell lines were selected and M345 (a combination of clones 123, 124 and 125), a sub-line of MCF7, which expressed the lowest levels of p21waf1 (Figure 6F), was the least inhibited by EB1089 (approximately 14%) as analyzed by crystal violet staining (Figure 6G). M124 cells, partially silenced for p21waf1 expression, were only partially inhibited up to 44%.

A recent report suggests that vitamin D_3_ and BRCA1 indirectly collaborate in regulation of 53BP1, a protein involved in DNA double strand break signaling and G2/M checkpoint activation [35]. In our study, no significant increase in 53BP1 expression level was observed by silencing of VDR or Brca1 (Figure 5A).

### BRCA1 and VDR are enriched in the chromatin fraction and their interaction is enhanced by EB1089

**Figure 7:**
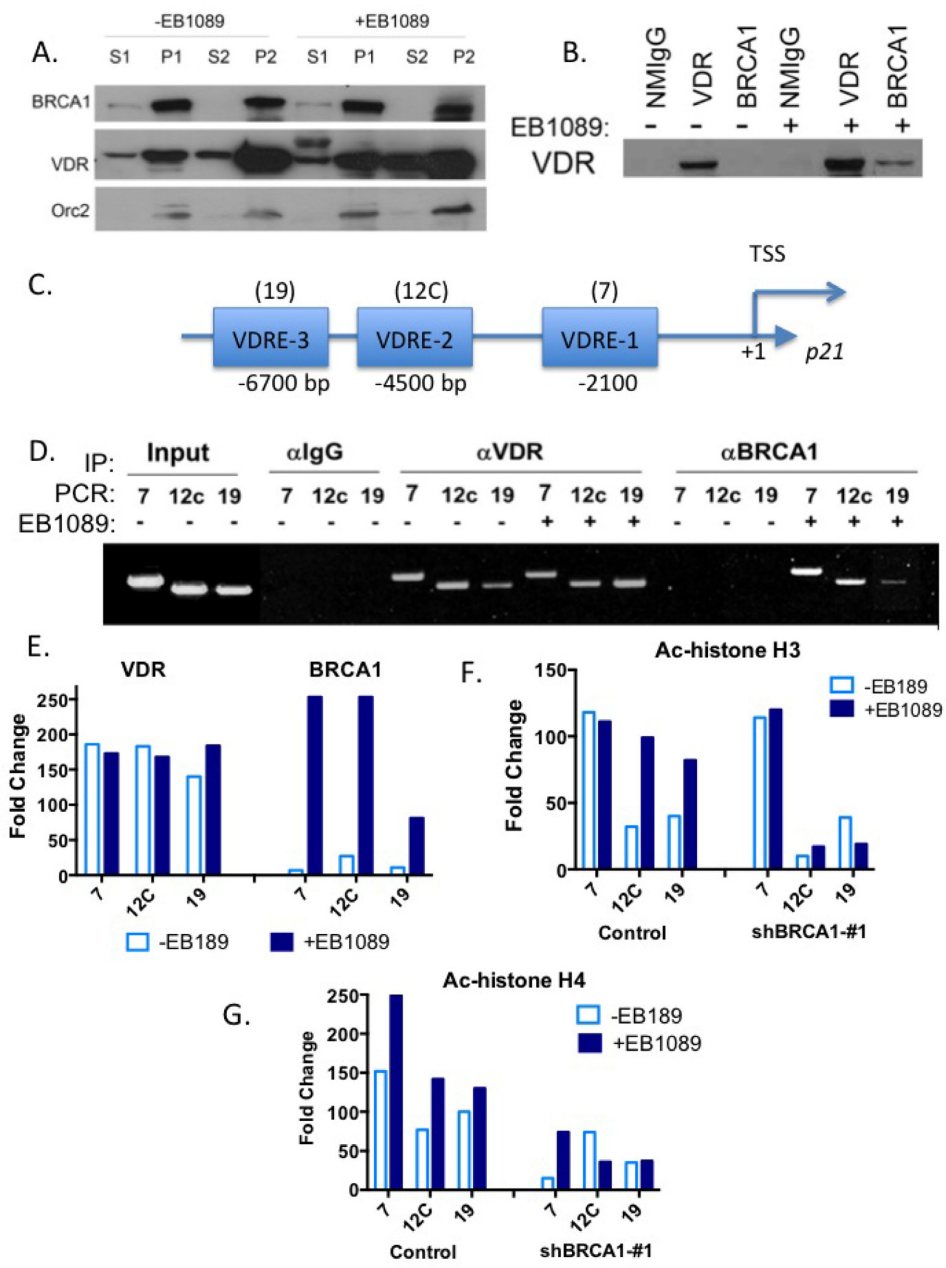
BRCA1 and VDR are in complex that co-occupies the promoter of p21waf1 promoter and up-regulates its expression **(A)**. MCF7 cells (1 × 10^7^ cells/treatment) were treated with vehicle or 1 μM of EB1089 for 1 hr and then cells were harvested and fractionated to the different cellular fractions as previously described [38, 61]. 100 μg of protein from each fraction were analyzed for BRCA1 and VDR expression by immunoblot analysis. Expression of Orc2 was analyzed as a positive control for nuclear and chromatin fractions. **(B)** MCF7 cells were treated with vehicle or 1 μM of EB1089 for 1 hr and cell whole extracts were prepared and immunoprecipitated with the indicated antibodies. Purified immunocomplexes were subjected to immunoblot analysis with VDR antibody. Normal mouse IgG served as a negative control. **(C)** Schematic representation of *CDKN1A* promoter with the specified locations of the three functional VDREs relative to transcription start site. **(D)** Chromatin was extracted from control and shRNA-expressing MCF7 cells after treatment with vehicle or 1 μM EB1089 for 1 hr. Chromatin was crosslinked, sheared by sonication and extracts were incubated overnight with anti-VDR or anti-BRCA1 antibodies and then the DNA-immunocomplexes were immunoprecipitated, washed extensively, and crosslinking was reversed. VDR and BRCA1 occupancy at the three functional VDREs present in the *CDKN1A* promoter was analyzed by PCR. NMIgG served as a control for specificity. The experiment was repeated three times independently and a representative gel is presented. **(E)** Densitometry of results in (D) were analyzed by Image J are presented in a bar graph. **(F-G)** Acetylation of three VDREs in the p21waf1 promoter in MCF7 cells in response to 1 μM EB1089 for 1 hr was analyzed by ChIP using anti-Ac-H3 (F) and anti-Ac-H4 antibodies (G). Results of three independent experiments are presented as bar graphs.

Given the nuclear localization of BRCA1, the enhanced translocation of VDR to the nucleus following vitamin D_3_, and that both BRCA1 and VDR associate with chromatin modifying enzymes [16, 36, 37], we examined whether BRCA1 and VDR are localized in the same cellular fraction. We fractionated EB1089-treated and vehicle-treated MCF7 cells by the centrifugation-based method developed by Mendez and Stillman [38] to cytoplasmic (S1), nuclear (P1) soluble nuclear (S2) or chromatin-enriched (P2) fractions. Immunoblot analysis demonstrated that BRCA1 and VDR were both detected in the chromatin-bound fraction of the nucleus and that treatment with EB1089 increased the chromatin-bound fraction of VDR (Figure 7A).

We next examined whether BRCA1 co-localization and cooperation with VDR is due to interaction between BRCA1 and VDR and whether the interaction is enhanced by vitamin D_3_. Co-immunoprecipitation assays suggested that endogenous BRCA1 and VDR are in a complex and the presence of a ligand enhances the interaction between the proteins in MCF7 and in HeLa cells (Figure 7B).

### BRCA1 and VDR co-occupy the p21 promoter at Vitamin D response elements

To determine whether BRCA1 and VDR spatially and temporally co-occupy vitamin D_3_ response elements (VDREs) in the *CDKN1A* promoter, we performed chromatin immunoprecipitation (ChIP) assays. The *CDKN1A* promoter contains multiple VDREs located within 7 Kb upstream of the transcriptional start site. Saramaki *et. al.,* [39] demonstrated that among all the putative VDREs present in the promoter, three are functional and activate transcription in response to vitamin D_3_ (Figure 7C). Not surprisingly, we found that VDR is bound to the three VDREs designated 7, 12c, and 19 [39], regardless of ligand treatment (Figure 7D and 7E). However, BRCA1 was only recruited to these three VDREs following treatment with EB1089 (Figure 7D and 7E). To determine whether BRCA1 recruitment changes the epigenetic landscape at the *CDKN1A* promoter, we analyzed histone acetylation marks that correlate with transcriptional activity by ChIP. Histone H3 acetylation at the VDRE sequence in region 7 probably is required for basal expression of p21waf1 as similar levels are detected in BRCA1-deficient and proficient cells regardless of EB1089 treatment. Histone H3 acetylation of 12C and 19 VDREs was significantly induced in BRCA1-proficient cells following EB1089 treatment and could barely be detected in BRCA1-deficient cells even in the presence of EB1089 (Figure 7F).

Acetylation of histone H4 at the vicinity of the three VDRE sequences of the *CDKN1A* promoter was significantly diminished in BRCA1-deficient cells and even in the presence of EB1089; histone H4 acetylation was not induced efficiently (Figure 7G). Our results suggest that vitamin D_3_, VDR and BRCA1 work in concert to modify the *CDKN1A* promoter landscape and to recruit histone-modifying enzymes that will facilitate induction of p21waf1 expression. In particularly, BRCA1 seems responsible for enrichment of H4 acetylation at the VDREs of *CDKN1* promoter.

## DISCUSSION

**Figure 8:**
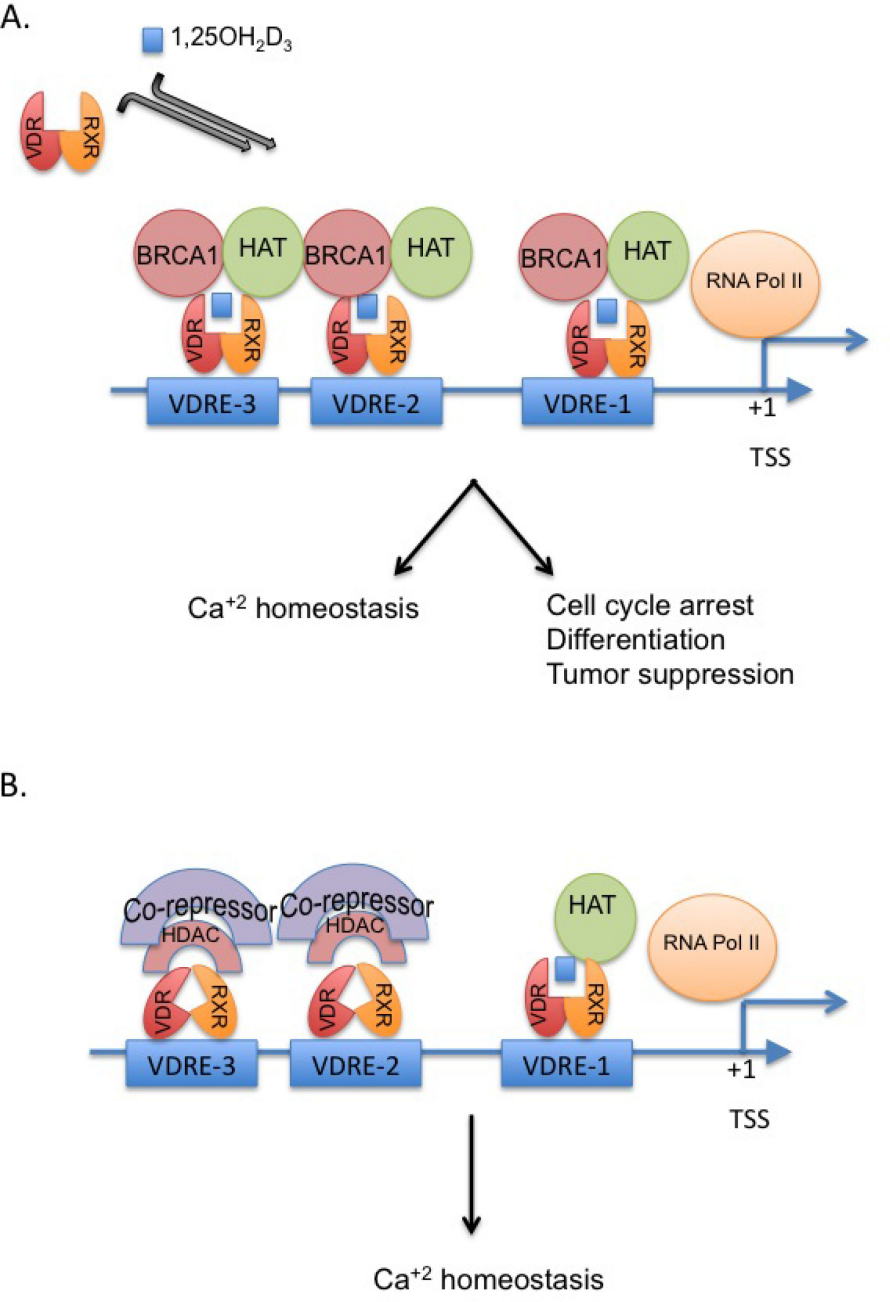
Proposed model for the crosstalk between BRCA1 and the vitamin D-VDR pathway The model displays the critical role of BRCA1 in vitamin D-mediated regulation of p21waf1 and growth inhibition **(A)**. In the absence of BRCA1, p21 as potentially other mutual targets of vitamin D and BRCA1 is not fully activated and cannot mediated cell cycle arrest and growth inhibition **(B)**.

BRCA1 and vitamin D_3_ are known for their anti-proliferative effects towards cancer cells. Both were previously linked to up-regulation of another tumor suppressor gene, *CDKN1A*, which encodes for the cell cycle G1 inhibitor, p21waf1 [34, 40]. Here we report for the first time that BRCA1 is critical for vitamin D_3_-mediated growth inhibition of breast cancer cells via co-regulation of cell cycle progression and p21waf1 expression (Figure 8). Although BRCA1-silenced MCF7 and MDA-MB-231 breast cancer cells are still responsive to vitamin D_3_, as reflected by Cyp24A1 mRNA induction, the G1/G0 checkpoint and p21waf1 induction were impaired despite similar expression leveles of vitamin D receptor (VDR) and its dimerization partner protein, RXR. In the absence of BRCA1 and vitamin D_3_, VDR was prominantly located in the cytoplasm as previously described [23]. However, upon vitamin D_3_ analogue (EB1089) treatment, VDR was still able to translocate into the nucleus in the majority of cells and was still transcriptionally active.

Interestingly, partially silenced MCF7 cells, which may model *BRCA1* haplosufficiency such as in heterozygote carriers with partial expression levels, exhibit an attenuated response to EB1089, suggesting that BRCA1 levels can modulate the extent of vitamin D_3_-mediated growth inhibition.

The cooperation between BRCA1 and vitamin D_3_ is maintained and even exaggerated in mammosphere cultures, which are enriched with stem-like, tumor initiating mammary cells. Our results show that mammospheres expressing the stemness marker ALDH1 [31], express comparable levels of VDR and RXR as their adherent counterparts and that vitamin D_3_ is still capable of inhibiting their growth and self-renewal. Importantly, tumor initiating cells and cancer stem cells are associated with resistance to chemotherapy, and are considered the precursors for recurrent tumors [27, 28]. Thus, the retained sensitivity of mammospheres to vitamin D_3_ inhibitory effects may have translational potential for breast cancer prevention and treatment. In contrast to our results, Pervin *et. al.,* recently suggested that mammosphere cultures are less sensitive to vitamin D_3_ [41]. In support of our results, So *et. al.* [42] reported that an analogue of vitamin D_3_ (BXL0124) represses the expression of the stem cell marker CD44 *in vitro* and also decreased tumor growth of a xenograft *in vivo* [42]. In addition, prostate cancer stem cells are also sensitive to the inhibitory effects of vitamin D_3_ [43]. Furthermore, our group and other groups examining, additional dietary compounds and natural metabolites including retinoic acid that signal via the nuclear hormone, RXR, that partners with VDR, find those natural metabolites particularly potent inhibitors of mammosphere cultures growth and self-renewal relative to adherent cells (manuscript submitted and [44–46]).

However, mammosphere cultures derived from BRCA1-silenced MCF7 or MDA-MB-231 breast cancer cells (enriched in ALDH1 expression) were no longer sensitive to the growth inhibitory effects of vitamin D_3_. Thus, our data suggest that vitamin D_3_-based therapies should take into account the genetic and genomic background of patients. *BRCA1* mutation carriers that express at least one wild type allele may still benefit from vitamin D_3_ as a preventive measure but patients with tumors null for BRCA1, will most likely be refractory to vitamin D_3_ growth inhibition.

There are multiple mechanisms that can lead to vitamin D_3_ resistance including loss of VDR expression, mutant forms of VDR [47], deregulated RXR phosphorylation [48], enhanced degradation of RXR [49] or increased expression of the protein, hnRNPA1, that binds VDRE sites with high affinity and prevents VDR-RXR binding [50]. Our data suggest that loss of BRCA1 expression results in partial vitamin D_3_ insensitivity. Although there was no significant loss of VDR or RXR expression, an aberrant localization of VDR was observed in BRCA1-depleted MCF7 cells as previously reported by Deng *et. al.,* [23] who hypothesized that proteasomal degradation of another unknown protein is involved in VDR mislocalization. Nevertheless, EB1089 treatment facilitated VDR translocation back into the nucleus in the majority of the cells and induced its transcriptional activity. The partial loss of vitamin D_3_-mediated growth inhibition is likely due to reduced transcriptional activity of growth inhibitory genes such as *CDKNA1* and potential loss of collaborative vitamin D-BRCA1 transrepression of proliferative genes.

We discovered a physical and functional interaction between BRCA1 and vitamin D transcriptional regulatory pathway. Both BRCA1 and VDR are enriched in the chromatin fraction (P2), especially in cells that were treated with vitamin D. BRCA1 interacts with VDR following EB1089 exposure and is tethered to regulatory regions that contain VDRE elements and augment transcription from those sites. Specifically, BRCA1 was recruited to the three functional VDRE sites in the *CDKN1* gene promoter at -2100 (region 7), -4500 (region 12C) and -6900 (region 19) bp relative to the transcription start site [39]. Luciferase activity confirmed the transcriptional role BRCA1 plays in augmenting p21/waf expression in response to vitamin D_3_ although the proximal promoter only contained region 7. Previous reports indicated that BRCA1 activates *CDKN1A* expression in both p53-dependent and p53-independent manner [14, 40]. Our cellular models consisting of ER positive MCF7 cells that harbor a wild type p53 gene, as well as, ER negative MDA-MB-231 cells that contain a mutant p53 gene, suggest that BRCA1-VDR cooperation is independent of p53. The role of ER, however, cannot be excluded as the culture media was depleted of steroids. BRCA1 is known to interact with ER and to inhibit its transcriptional activity [51]. It is possible that in the presence of vitamin D_3_, VDR displaces ER. BRCA1 co-occupation of VDREs following ligand binding suggests that BRCA1 is a transcriptional co-activator of VDR that may assist in recruitment of additional chromatin modifying enzymes. Previously, we reported that BRCA1 interacts with histone deacetylase enzymes 1 and 2 (HDAC1 and HDAC2) [16], while Pao *et. al.,* [52] reported that BRCA1 interacts with the transcriptional co-activator, CBP/p300 to catalyze protein and histone acetylation. The interaction of BRCA1 with chromatin modifying enzymes with opposing activities is likely promoter and context dependent; BRCA1 may interact with factors that induce chromatin de-condensation and a permissive landscape when it associates with VDR to up-regulate the expression of p21waf1 and other growth inhibitory genes or it may form complexes that induce condensed, non-permissive chromatin to negatively regulate the expression of genes involved in cell proliferation as ER or PCNA [53–55].

Histone acetylation is associated with active chromatin. In the absence of BRCA1 expression, the VDREs *in the CDKN1A* promoter are decorated with less acetylated histones, especially acetylated histone H4, which potentially impairs the response to EB1089. BRCA1 expression also impacts the level of H3 acetylation at the remote VDREs but does not seem to play a major role in the acetylation of the VDRE that is located closest to the transcription start site (region 7). While it is unclear which modifying enzyme was lost or has failed to be recruited due to loss of BRCA1 expression, Malinen *et. al.,* [56] reported that induction of p21waf1 expression in MCF7 cells is attenuated by interaction between VDR and HDAC3 and HDAC7. Thus, it can be speculated that BRCA1 and VDR association forms after or leads to HDAC3 and HDAC7 displacement. Alternatively, BRCA1 may be recruited for activation of VDR target genes for its E3 ubiquitin ligase activity [57]. Recently it was shown that BRCA1 ubiquitinates histone H2B, a modification that is tightly associated with enhanced transcription [58]. However, no association between VDR and histone H2B ubiquitination has been reported thus far.

This is the first report that links BRCA1 to cell cycle arrest at the G1/G0 phase without exposure to DNA damage. It will be interesting to test the interaction between BRCA1 and VDR to see whether they continue to cooperate in response to DNA damage.

The current paradigm of successful breast cancer therapy insists on coordinated elimination of highly proliferative, therapy-sensitive breast tumor cells as well as of dormant breast cancer stem cells that are often resistant to conventional chemotherapy regiments. Here we show that vitamin D_3_ –based therapies could potently target those dormant cells in combination with other cytotoxic therapies, but will only benefit patient who express some level of BRCA1. Thus, the vision of effective vitamin D_3_-based therapies or prevention needs to take into account the genetic background of individual patients.

## MATERIALS AND METHODS

### Cell culture and reagents

MCF7, MDA-MB-231, HCC1937 and HeLa cells were obtained from ATCC and grown in Dulbecco modified Eagle medium containing 10% heat inactivated fetal bovine serum (Gibco). HEK293T cells were grown in RPMI 1640. WT145 and KO240 cells [a kind gift of J Welsh U. Albany NY) were described elsewhere [32] and were grown in DMEM/Ham's F12 (Gibco) containing 10% heat inactivated fetal bovine serum (Gibco). Cells were maintained as monolayers in a humidified atmosphere containing 5% CO_2_ at 37°C. EB1089 (Seocalcitol) was kindly provided by Leo Pharmaceutical Ltd (NL) and later obtained from Tocris. Prior to treatments with EB1089 (dissolved in EtOH), cells were washed twice with 1 × PBS and the growth medium was replaced with phenol-red-free fresh DMEM (Gibco) that contained 10% heat-inactivated, charcoal-treated FBS (Gibco).

### Plasmids, transfections and gene silencing by shRNA

Human BRCA1 expression was silenced by stable infection with a cocktail of On-TARGET SMART pool pLKO.1-lentiviral shRNAs corresponding to *BRCA1* sequences (Supplementary table 1) obtained from Open Biosystems (ThermoFisher Inc.) Lentiviral plasmids were transfected into HEK293T for 48 hr. Virions were collected, filtered, and transduced into MCF7 or MDA-MB-231 Cells with 8 μg/ml Hexadimethrine bromide (polybrene, Sigma). Puromycin selection (2 μg/ml for MCF7 and 0.5 μg/ml for MDA-MB-231) began 48 hr post infection. After 14 days, puromycin-resistant colonies were isolated and expanded to generate stable BRCA1-silenced MCF7 and MDA-MB-231 sublines. Human p21waf1 expression was stably silenced with a cocktail of On-TARGET SMART pool pLKO.1-lentiviral shRNA corresponding to *CDKN1A* gene (Supplementary table 1) as described above (Open Biosystems, ThermoFisher Inc.). Murine Brca1 expression was transiently silenced with siGENOME SMARTpool (Open Biosystems, ThermoFisher Inc.). Ad-BRCA1 and Ad-vecctor were previously described [20].

### Mammosphere formation and Self-renewal assays

Mammosphere cultures derived from MCF7 and MDA-MB-231 cells were formed as previously described [30, 46]. Briefly, MCF7 single cell suspension was diluted to a concentration of 10,000 cell/ml in serum-free phenol-red free MEBM (MEGM Bulletkit, Lonza) supplemented with 5 μg/ml bovine insulin, 20 ng/ml recombinant epidermal growth factor (Peprotech), 20 ng/ml basic fibroblast growth factor (Peprotech), 1 × B27 supplement (Gibco), 0.5 μg/ml hydrocortisone (Sigma) and plated into low attachment plates. Self-renewal capacity of the mammospheres was determined by dissociation, re-plating and producing further generations. For secondary and tertiary mammospheres, dissociated cells were replated at 1,000 cells/ml. MDA-MB-231 single cell suspension at a concentration of 60,000 cell/ml in serum-free phenol red-free CnT-27 medium (CellnTEC Advanced cell systems, Bern, Switzerland) supplemented with growth additives as described [59] was plated into low attachment plates. Media was replenished every 3–4 days. For secondary mammospheres a single cell suspension at a concentration of 3000 cells/ml was plated into low attachment plates.

### Crystal violet growth assays

Cells were seeded at 1500 (MDA-MB-231) or 4000 cells (MCF7, HCC1937) per well of 96 well plates in 6 replicates. On the following day, the media was replaced with phenol-free DMEM supplemented with 10% charcoal-treated FBS and the indicated doses of EB1089 or EtOH as vehicle control. At the indicated time points, individual plates were fixed and stained with crystal violet solution as previously described [46]. The results are expressed as the percentage of controls following calculation of the replicate's mean (± SD) from three independent experiments.

### XTT viability assay

Cells were seeded into a 96 well plates at 1500 cells per well (MDA-MB-231) or 4000 cells (MCF-7) per well in triplicate in normal growing media. On the following day, the media was replaced with phenol-free DMEM supplemented with 10% charcoal-treated FBS and the indicated concentrations of EB1089. Cells were incubated for the indicated times, at which time XTT (2, 3,-bis (2-methoxy-4-nitro-5-sulfophenyl)-5-[(phenylamino)-carbonyl]-2H-tetrazolium inner salt) reduction was used to quantify viability according to manufacturer's instruction (ATCC). Absorbance was recorded by a photometer SPEKTRAFluor Plus, Tecan (Salzburg, Austria) at 450 nm with 650 nm of reference wavelength. Cell survival was estimated from the equation: % cell survival = 100 × (At-Ac), where At and Ac are the absorbencies (450nm and 650 nm respectively) of the XTT colorimetric reaction (ATCC) in treated and control cultures. Absorbance of medium alone was also deducted. The results are expressed as the percentage of controls following calculation of the replicate's mean (± SD) from three experiments.

### Aldefluor assay and flow cytometry

ALDH1 activity was measured with the Aldefluor kit according to manufacturer's instructions (Stem Cell Technologies). Briefly, MCF-7 and MDA-MB-231-derived mammospheres were trypsinized, gently vortexed and passed through a 40μM cell filter to produce single cell suspensions. Cells (5 × 10^5^) were washed once and then resuspended in Aldefluor assay buffer containing uncharged ALDH1-substrate, BODIPY-aminoacetaldehyde (BAAA), and incubated for 45 min at 37°C, with gently vortexing every 15 mins. Fluorescent ALDH1-expressing cells were detected in the green fluorescence channel (520–540 nm) of a FACScan instrument (BD Biosciences). A second set of cells under identical conditions, were stained with the specific ALDH inhibitor, diethylaminobenzaldehyde (DEAB), to serve as a negative control for the experiment. Cells incubated with BAAA and DEAB were used to establish the baseline fluorescence of cells and ALDH1-positive fraction. Data were analyzed by using Cell Quest software (BD Biosciences).

For cell cycle analysis, cells (1 × 10^6^) were washed twice with 1 × PBS (pH 7.4), centrifuged at 360g for 5 min at 4°C, and fixed in chilled ethanol (70%; v/v in PBS) with gentle vortex mixing and incubated at least for over night at −20°C. To determine the DNA contents, the cells were stained with 40 μg/ml propidium iodide (PI, BD Biosciences) and analyzed using a FACSCalibur flow cytometer and analyzed using CellQuest analysis software (BD Biosciences).

### Immunoblots and immunoprecipitations

Protocols for these procedures have been previously described [57]. The following primary antibodies were used: human BRCA1 (Ab-1, Calbiochem OP92 and D-9, Santa Cruz sc-6954), murine Brca1 [60], human and murine VDR (D-6; Santa Cruz sc-13133), RXR (D-20; Santa Cruz sc-553), p21 (AB-1; Calbiochem), p27 (Santa Cruz sc-1641), 53BP1 (Bethyl A300-272A), GAPDH (FL-335; Santa Cruz sc-25778), ⍺-Tubulin (Sigma, T9026) and Orc2 (H300; Santa Cruz sc-28742).

### Immunofluorescence

Cells were plated onto coverslips, fixed with 4% (w/v) paraformaldehyde in PBS for 15 min and then premeabalized with 0.5% Triton X-100 for 5 min. cells were washed twice with 1 × PBS and incubated with 10% goat serum for 1 hr at room temperature. Cells were incubated with primary antibodies: BRCA1 (AB1, Calbiochem and VDR, Santa Cruz) for 1 hr at 37°C. Cells were washed three times with 1 × PBS and protein localization was visualized with secondary antibodies Rabbit anti Mouse IgG-conjugated with Alexa 488 and Goat anti Rabbit IgG-conjugated with Alexa 594 (Life Technologies) under the Leica TCS SP5 II microscope (The Sackler Cellular & Molecular Imaging Center (SCMIC)).

### Dual iuciferase assays

Cells were transfected with 4 μg of the pWW-Luc construct or 2 μg of the pRL-3 Renilla vector with Lipofectamine 2000 according to manufacturer's instructions (Life Technologies). On the following day, the culture media was replaced with phenol-red free DMEM with 10% charcoal-treated FBS. Cells were treated with 1 μM EB1089 or vehicle and harvested 24 hr later. Cell lysates were assayed with the Dual Luciferase Assay (Promega, WI, USA), according to manufacturer's instructions. Firefly luciferase values were normalized to the Renilla signal, and the ratio of the Firefly/Renilla values were reported. All of the transfection assays were carried out in triplicates.

### Chromatin fractionation

Chromatin fractionation was performed as described [38, 61]. The preparation was carried from duplicate samples to enable presentation of all fractions. Approximately 1 × 10^7^ cells were washed in PBS and resuspended in 200 μl of solution A (10 mM Hepes [pH 7.9], 10 mM KCl, 1.5 mM MgCl_2_, 0.34 M sucrose, 10% glycerol, 1 mM DTT, and protease and phosphatase inhibitors). Triton X-100 was added to a final concentration of 0.1%, cells were incubated on ice for 5 min, and the cytoplasmic (S1) and nuclear fractions (P1) were harvested by centrifugation at 1,300 × g for 4 min. Isolated nuclei were then washed in solution A, lysed in 150 μl solution B (3 mM EDTA, 0.2 mM EGTA, 1 mM DTT, and protease and phosphatase inhibitors), and incubated on ice for 10 min. The soluble nuclear (S2) and chromatin fractions (p2) were harvested by centrifugation at 1,700 × g for 4 min. Isolated chromatin was washed once with solution B and spun down at high speed (10,000 × *g* for 1 min). Finally, chromatin was resuspended in 150 μl of SDS sample buffer and sheared by sonication. Protein concentrations were determined by BCA assay (ThermoScientific, Rockford, IL). Equal amounts of protein from whole cell extracts or from the different cellular fractions were mixed with 5 × laemmli buffer (Bio-Rad) and analyzed by immunobloting.

### Chromatin immunoprecipitation

Chromatin Immunoprecipitation (ChIP) was performed according to ChIP Assay Kit (EMD-Millipore). MCF7 cells (1 × 10^7^/treatment) were seeded and on the following day, the growing medium was replaced with phenol red free-DMEM supplemented with 10% charcoal–treated FBS. On the next day, cells were treated for 1 hr with EB1089 (1 μM) or vehicle as a control. Prior to lysis, cells were crosslinked with 1% formaldehyde for of 10 min at 37ºC. Crosslinking was quenched by adding glycine to a final concentration of 0.125 M. The cells were lysed in cold SDS Lysis Buffer (600 μl, ChIP kit-Millipore) and incubated for 10 min on ice. Lysates were sonicated to shear the DNA (10 seconds twice at 30% of max sonication output). The samples were centrifuged for 10 min at 13,000 rpm at 4°C, and the sonicated cell supernatants were diluted tenfold in ChIP Dilution Buffer (ChIP kit-Millipore), supplemented with protease inhibitors as above. Chromatin (200 μg/sample) was precleared with Salmon Sperm DNA/Protein A Agarose- for 30 min at 4°C and then incubated over night with the following antibodies: IgG (Santa Cruz sc20265), acetylated Histone 4 (EMD-millipore 06-598), acetylated Histone 3 (EMD-Millipore 17-10051), BRCA1 (Ab-1 from Calbiochem or sc-6954 from Santa Cruz) and VDR (Santa Cruz sc-1008). DNA was eluted from the recovered protein-DNA immunocomplexes and following removal of the crosslinked proteins, PCR amplifications of the three VDRE sequences within the *CDKN1A* promoter were performed with Platinum PCR supermix (Life Technologies). Primer sequences were as previously reported [39]. For fragment 7: F 5′-CACCACTGAGCCTTCCTCAC and R 5′-CTCGACTCCCAGCACACACTC; for fragment 12C: F 5′-CGCGGTGCTTGGTCTCTATG and R 5′-CCTTTCCCAACAAACAAGGGG; for fragment 19: F 5-CTAACCTCACAGTACAGGCC and R 5′-GCCTCTTTGTGCCTTTGCAC.

### RNA isolation and and RT-PCR analysis

Total RNA was isolated from cells using TRIZOL (Life Technologies). cDNA was synthesized using Superscript II according to manufacturer's instructions (Life Technologies). Real-time PCR of samples was done using an ABI7900HT sequence detection system (Applied Biosystems) in the presence of SYBR green (Qiagen). Experiments were performed in triplicate, data were normalized to the housekeeping gene, GAPDH, and the relative abundance of transcripts was calculated by the comparative ΔΔ*Ct* method. All primer sequences are available in the Supplementary Materials and Methods section.

### Statistical analysis

Quantitative data is expressed as mean ±SD and differences were compared using two-tailed Student *t*-test. The Pearson product-moment correlation coefficient test was performed to test the correlation of two sets of results. Statistical analysis was carried out in GraphPad, PRISM. *P* values less than 0.05 were considered statistically significant.

## SUPPLEMENTARY TABLES AND FIGUERS



## Abbreveations

BRCT: BRCA1 carboxyl terminus
ChIP: chromatin immunoprecipitation
ER: estrogen receptor
EtOH: ethyl alcohol
FBS: Fetal Bovine Serum
HDAC: histone deacetylase
hr: hours
mRNA: messenger RNA
NMIgG: Normal mouse Immunoglobulin G
PBS: phosphate buffer saline
qRT-PCR: quantitative real-time PCR
RXR: retinoid-x receptor
shRNA: short hairpin RNA
siRNA: short interfering RNA
VDR: vitamin D receptor
VDRE: vitamin d response element
Vitamin D_3_: 1,25-dihydroxyvitamin D_3_ [1,25(OH)_2_D3]
