# Supplementary material for: Cooperation between BRCA1 and vitamin D is critical for histone acetylation of the p21waf1 promoter and for growth inhibition of breast cancer cells and cancer stem-like cells
Source: Oncotarget. 2014 Nov 26;5(23):11827–46. doi: 10.18632/oncotarget.2582 (PMC4322975; doi:10.18632/oncotarget.2582)
Supplement: Supplementary file 1 [file oncotarget-05-11827-s001.pdf]

SUPPLEMENTARY TABLES AND FIGUERS

Supplementary Table S1. BRCA1 shRNA targeting sequences

Clone Id	Mature targeting sequence
	5′-AGTTTCGTTGCCTCTGAAC
9823	5′-ATTCATGCCAGAGGTCTTATA
9824	5′-CTAGTCCTTCCAACAGCTATA
10305	5′-TTCAGTATCTCTAGGATTCT
39833	5′-TTTAGAGAAGTAAACTTAGGG
39834	5′-ATTCAGTACAATTAGGTGGGC
39835	5′-AATTCAGTACAATTAGGTGGG
39836	5′-AATGATGGGCATTTAGAAGGG
39837	5′-AAACCCAGGGCTGCCTTGGAAAAG

Supplementary Table S2. CDKNA1 shRNA targeting sequences

Clone Id	Mature targeting sequence
123	5′-TAAGGCAGAAGATGTAGAGCG
124	5′-TTCCTCTTGGAGAAGATCAGC
125	5′-AAAGTCGAAGTTCCATCGCTC
126	5′-TTGGAGTGGTAGAAATCTGTC
127	5′-AAACCCAGGGCTGCCTTGGAAAAG
400	5′-TTCCTCTTGGAGAAGATCAG
401	5′-CCAGCACTCTTAGGAACCTCT

Supplementary Table S3. qRT-PCR primers

Gene	Forward	Reverse
BRCA1	5′-TGGAAGAAACCACCAAGGTC	5′ ATGGAAGCCATTGTCCTCTG
VDR	5′-TGGCTTTCACTTCAATGCTATGA	5′-CGTCGGTTGTCCTTGGTGAT
CDKN1A	5′ CCTCATCCCGTGTTCTCCTTT	5′ GTACCACCCAGCCAAGT
CYP24A1	5′ GACTACCGCAAAGAAGGCTAC	5′ CATCACTTCCCCTGGTTTCATTA
Notch1	5′ CGGGTCCACCAGTTTCAATG	5′ GTTGTATTGGTTCGGCACCAT
KRT18	5′ CCTGTCCTTTCTCTCTCCCC	5′ CTCCTTCTCGTTCTGGATGC
GAPDH	5′ TGGACCTCATGGCCCACA	5′ TCAAGGGGTCTACATGGCAA
β-Actin	5′ CCTGGCACCCAGCACAAT	5′ GCCGATCCACACGGAGTACT

Supplementary Table S4. Lentivirus Silencing vectors

A

Clone ID number	Our number
39833	1
39837	2
39834	3
10305	4
39835	5
9823	6
39836	7
9824	8

B

Clones number	Combination number
1.2.3	1
2.4.5	2
5.7.8	3
1.8.5 (only MCF7)	4
1.3.8	6
4.7	7

BRCA1 expression was silenced by stable infection with lentiviral shRNA corresponding to BRCA1 sequence (Open Biosystems, Thermofisher Inc.). The viral vectors are based on the pLKO.1 plasmids. The kit contains 8 different shRNA plasmids sequences based on the RNAi consortium (TRC). Different combinations of the eight lentiviral shRNA vectors were used to achieve maximum silencing.

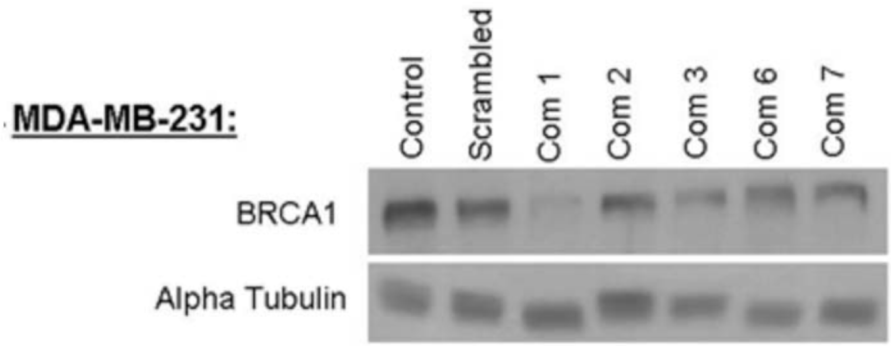

Supplementary Figure S1: shRNA targeting of BRCA1 in MDA-MB-231. MDA-MB-231 cells were stably infected with a cocktail of SMARTpool shBRCA1 or with shScrambled sequence of BRCA1. Puromycin –resistance colonies were expanded and 100 µg of whole cell lysates from the newly formed sublines were analyzed for BRCA1 expression by immunoblot analysis. Com- combination of silencing vectors as shown in Supplementary Table 4.

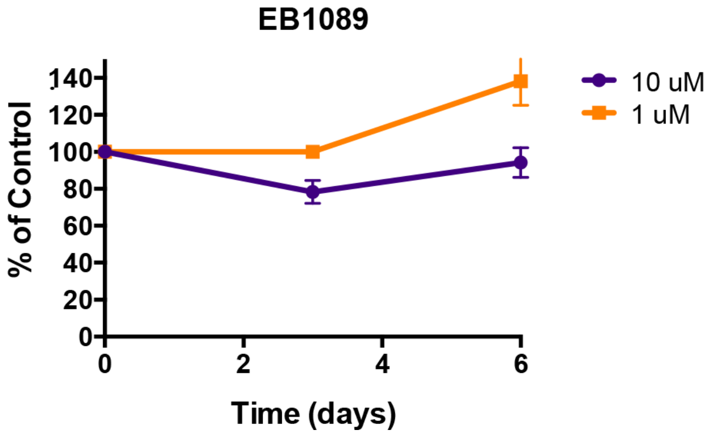

Supplementary Figure S2: EB1089 does not inhibit growth of BRCA1-silenced MDA-MB-231 cells. BRCA1-deficient and proficient MDA-MB-231 cells were seeded into 96 well plates (1500 cells/well) and their growth in response to the indicated concentrations of EB1089 was analyzed by crystal violet assay. Values represent the percent of control, vehicle treated cells. Graph is a representative of the mean ± SD of 3 replicates in two independent experiments.

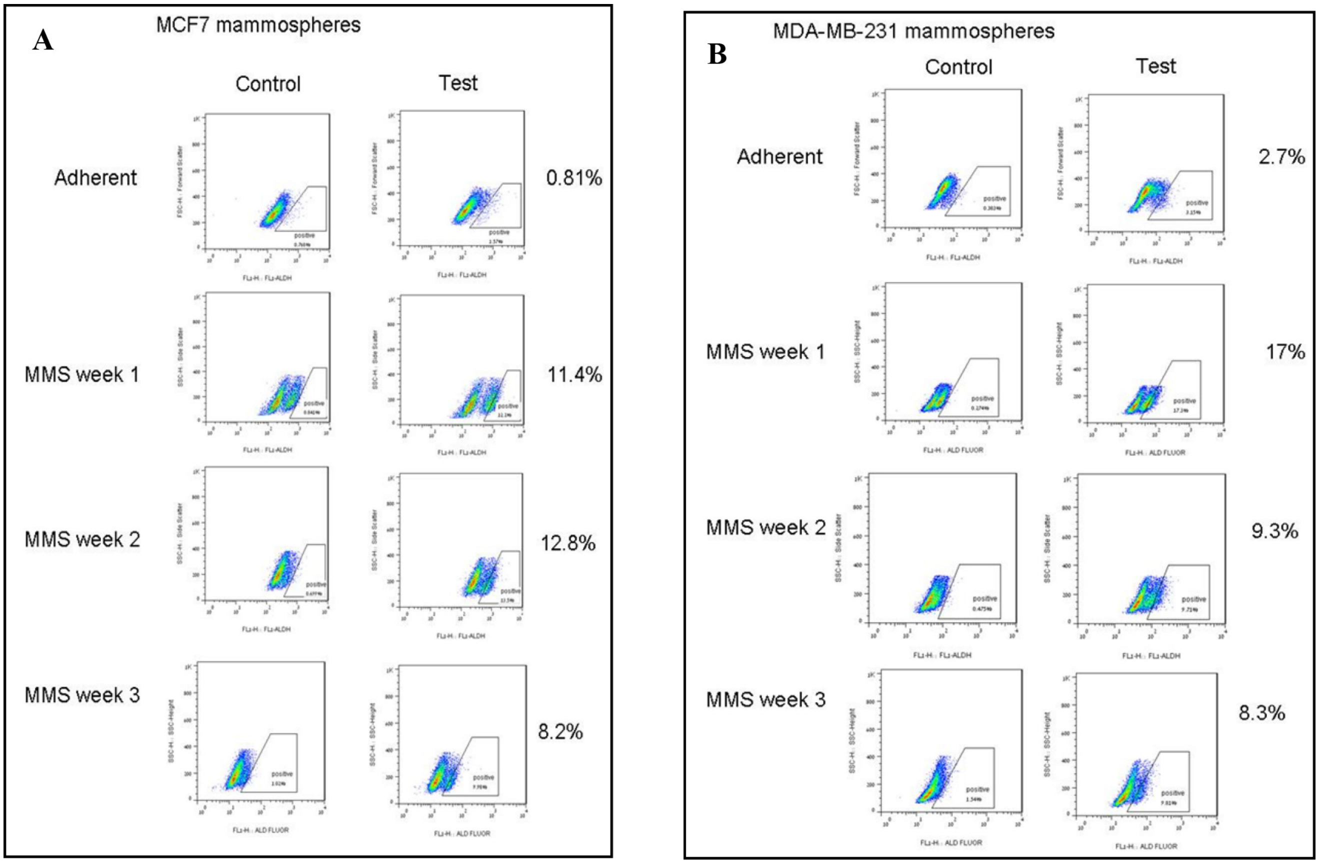

Supplementary Figure S3: ALDH1 activity in MCF7 and MDA-MB-231 –derived mammospheres. MCF7 **(A)** and MDA-MB-231 **(B)** adherent and MMS cultures collected, and following dissociation into single cells suspension, cells were incubated with ALDEFLUOR reagent or with DEAB (a specific inhibitor of ALDH). After 1 hour incubation, the cells were washed and analyzed by flow cytometry.

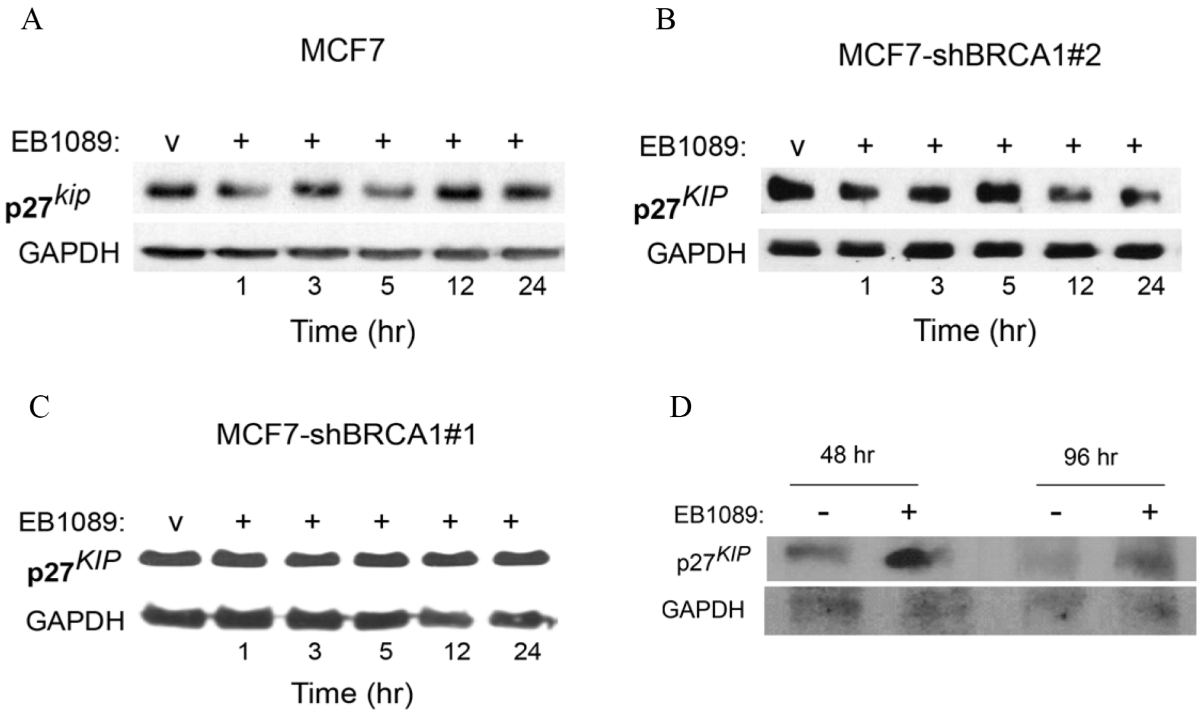

Supplementary Figure S4: P27Kip is not regulated by BRCA1 and vitamin D directly. MCF7 control **(A)** and MCF7 stably expressing BRCA1-targeting shRNAs: **(B)** shBRCA1-#2-MCF7 and **(C)** shBRCA1-#1-MCF7 were treated with vehicle or 1¼M EB1089 for the indicated times. Whole cell lysates were prepared and subjected to immunoblot analysis p27kip expression. **(D)** MCF7 cells were treated with vehicle or 1¼M EB1089 for the longer times as indicated and subjected to immunoblot analysis for expression of p27kip. GAPDH served as a control.
